# Changes in Stress, Anxiety, and Depression Levels of Subscribers to a Daily Supportive Text Message Program (Text4Hope) During the COVID-19 Pandemic: Cross-Sectional Survey Study

**DOI:** 10.2196/22423

**Published:** 2020-12-18

**Authors:** Vincent Israel Ouoku Agyapong, Marianne Hrabok, Wesley Vuong, Reham Shalaby, Jasmine Marie Noble, April Gusnowski, Kelly J Mrklas, Daniel Li, Liana Urichuk, Mark Snaterse, Shireen Surood, Bo Cao, Xin-Min Li, Russell Greiner, Andrew James Greenshaw

**Affiliations:** 1 Department of Psychiatry Faculty of Medicine and Dentistry University of Alberta Edmonton, AB Canada; 2 University of Calgary Calgary, AB Canada; 3 Alberta Health Services Edmonton, AB Canada; 4 Provincial Clinical Excellence Alberta Health Services Calgary, AB Canada; 5 Department of Community Health Sciences Cumming School of Medicine University of Calgary Calgary, AB Canada

**Keywords:** COVID-19, mobile technology, text, anxiety, depression, stress, outbreak, pandemic, mental health, outreach

## Abstract

**Background:**

In addition to the obvious physical medical impact of COVID-19, the disease poses evident threats to people’s mental health, psychological safety, and well-being. Provision of support for these challenges is complicated by the high number of people requiring support and the need to maintain physical distancing. Text4Hope, a daily supportive SMS text messaging program, was launched in Canada to mitigate the negative mental health impacts of the pandemic among Canadians.

**Objective:**

This paper describes the changes in the stress, anxiety, and depression levels of subscribers to the Text4Hope program after 6 weeks of exposure to daily supportive SMS text messages.

**Methods:**

We used self-administered, empirically supported web-based questionnaires to assess the demographic and clinical characteristics of Text4Hope subscribers. Perceived stress, anxiety, and depression were measured with the 10-Item Perceived Stress Scale (PSS-10), the Generalized Anxiety Disorder–7 (GAD-7) scale, and the Patient Health Questionnaire–9 (PHQ-9) scale at baseline and sixth week time points. Moderate or high perceived stress, likely generalized anxiety disorder, and likely major depressive disorder were assessed using cutoff scores of ≥14 for the PSS-10, ≥10 for the GAD-7, and ≥10 for the PHQ-9, respectively. At 6 weeks into the program, 766 participants had completed the questionnaires at both time points.

**Results:**

At the 6-week time point, there were statistically significant reductions in mean scores on the PSS-10 and GAD-7 scales but not on the PHQ-9 scale. Effect sizes were small overall. There were statistically significant reductions in the prevalence rates of moderate or high stress and likely generalized anxiety disorder but not likely major depressive disorder for the group that completed both the baseline and 6-week assessments. The largest reductions in mean scores and prevalence rates were for anxiety (18.7% and 13.5%, respectively).

**Conclusions:**

Text4Hope is a convenient, cost-effective, and accessible means of implementing a population-level psychological intervention. This service demonstrated significant reductions in anxiety and stress levels during the COVID-19 pandemic and could be used as a population-level mental health intervention during natural disasters and other emergencies.

**International Registered Report Identifier (IRRID):**

RR2-10.2196/19292

## Introduction

COVID-19, an acute respiratory disease, was first reported in December 2019 in Wuhan, China. Since the outbreak was declared a pandemic by the World Health Organization [1,2], this disease has continued to have significant, unprecedented impacts on health and patterns of human life worldwide. These impacts include school and business closures as well as the ongoing psychological and social tolls of uncertainty, vigilance, and quarantine. In addition to the obvious physical medical impact of this disease [3,4], it poses evident threats to people’s mental health, psychological safety, and well-being [5-7], particularly given the risk of recurrent outbreaks [8].

In multiple global jurisdictions, a series of mental health concerns have arisen, including increased stress, anxiety, depression, fear, insomnia, and obsessive-compulsive behaviors. Population-level studies have summarized these effects [9,10]. For example, in a study in China, over half of the respondents rated the psychological impact of COVID-19 as moderate or severe, with 29% reporting significant anxiety symptoms and 17% reporting significant depressive symptoms [11].

The emergence of mental health issues during the COVID-19 pandemic was not entirely unexpected. There have been reports of increases in stress symptoms, confusion, anger, anxiety, and depression [12-15] as well as in problematic drug and alcohol use [7] related to previous pandemics. Stressors include long quarantine durations, infection fears, frustration, boredom, inadequate supplies, inadequate information, financial loss, and stigma. Quarantine, in particular, is associated with a number of negative psychological and social effects (eg, posttraumatic stress, anger, fear, financial loss, and stigma) [12].

Although research has provided a description of the psychological impact of COVID-19 [16], the literature regarding interventions or guidelines for managing the mental health impacts of the virus is limited [17]. Countries that were impacted initially by the COVID-19 pandemic identified several problems that increased the difficulty of providing psychological interventions during the pandemic, including barriers to participation, limited efficiency of outreach, and limited capacity of frontline workers to provide support due to competing demands on their time and energy [18]. Provision of psychological support during this pandemic is further complicated by the high number of people requiring support and the need to maintain physical distancing.

The COVID-19 pandemic has further reinforced the need and urgency of transforming the delivery of mental health services [19] to include telehealth, text messaging, and other digital platforms. Mobile health technologies offer a unique and innovative solution in this context. More specifically, SMS text messaging via mobile phones offers a convenient, cost-effective, and accessible means of implementing population-level interventions. In Canada, almost 90% of residents own a smartphone [20]. Additionally, SMS text messaging is embedded in 98% of mobile phones [21]. Texting is free to the majority of end users, does not require technical skill to use, and is included in most mobile plans. SMS text messages are also cost-effective for providers [22].

Previous research examining the effectiveness of supportive text messages has demonstrated positive outcomes, including reduction of depressive symptoms and high user satisfaction [23-25]. For example, evaluation of Text4Mood, a text messaging intervention administered following large-scale forest fires in Fort McMurray, Alberta, found that supportive text messages helped subscribers feel more hopeful about managing issues (82%), in charge of managing their depression and anxiety (77%), and connected to a support system (75%); moreover, subscribers stated that the intervention improved their overall mental well-being (83%) [24]. Similar findings were observed in other studies, including Text4Baby, which sought to assist women by providing supportive and informative text messaging during pregnancy, and another text intervention aimed to support the mental health of impoverished women in Bangalore. Participants in both interventions indicated that receiving these text messages gave them a sense of reassurance and made them feel supported [26].

On March 23, 2020, Alberta Health Services, along with the coauthors of this paper, initiated Text4Hope, a 3-month-long, supportive daily text messaging program using principles of cognitive behavioral therapy (CBT), as an additional mental health support for people living in Alberta during the COVID-19 pandemic [27]. The messages ultimately seek to reduce or inhibit negative thought patterns while suggesting and reinforcing the use of healthy self-coping mechanisms. This program was intended to complement existing addiction and mental health services that individuals might be accessing at the time of participation.

This paper evaluates the impact of Text4Hope on measures of stress, anxiety, and depression symptoms and provides estimates of prevalence rates 6 weeks into the program.

## Methods

### The Text4Hope Program

This cross-sectional comparative study sought to assess the effectiveness of community implementation of a supportive SMS text message intervention program focused on reducing symptoms of stress, anxiety, and depression during the COVID-19 pandemic. The study protocol [28] was approved by the Research and Ethics Board of the University of Alberta (Pro00086163).

In the Text4Hope program [29], individuals self-subscribe to receive daily supportive SMS text messages for three months by texting the word “COVID19HOPE” to a short code number. The messages are aligned with a cognitive behavioral framework, with content written by mental health professionals and coauthors of this paper (VIOA, MH). The messages were uploaded to a web-based platform, which delivered messages at 9 AM each day. The first message welcomed subscribers to the service and invited them to complete a web-based baseline survey that captured demographic and clinical information. At 6 weeks, subscribers were invited again via a text message link to complete a web-based follow-up survey. At baseline and at 6 weeks, we collected clinical information on stress, anxiety, and depression about each subject based on the 10-Item Perceived Stress Scale (PSS-10) [30], Generalized Anxiety Disorder-7 (GAD-7) scale [31], and the Patient Health Questionnaire-9 (PHQ-9) [32], respectively.

### Data Collection

**Figure 1:**
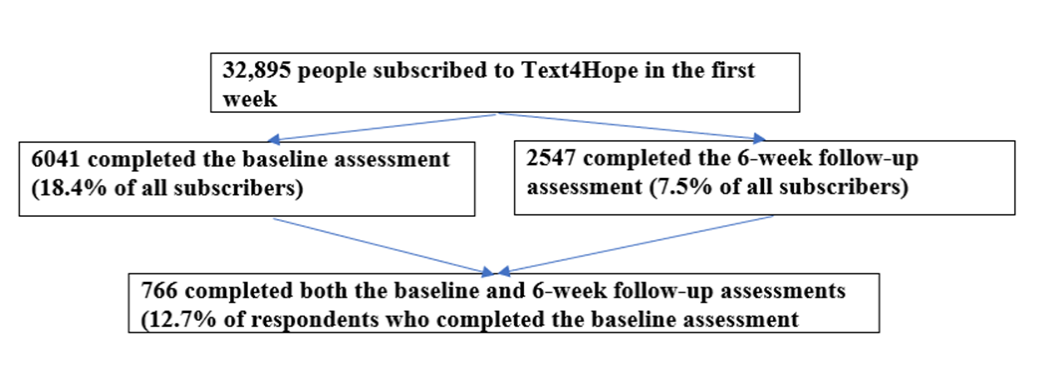
Flowchart of subscriber participation from baseline to the sixth week.

We were able to cross-reference clinical and demographic responses from individuals by asking clients to enter the mobile number they used for Text4Hope at the baseline and 6-week time points. No incentives were offered to respondents. Participation in the program was voluntary, and completing the survey was not required to receive the supportive SMS text messages. Subscribers could opt out at any time by texting “STOP” to the same sort code number used to enroll in the program. Baseline data collection occurred between March 23 and 30, 2020, and the sixth week follow-up data were collected between May 3 and 11, 2020. Figure 1 presents the subscriber flowchart, which indicates the number of subscribers who completed the web-based surveys at each time point. Furthermore, on May 11, 2020, there were 45,775 subscribers to the Text4Hope program, of which 6178 subscribers opted out of the program, giving a dropout rate of 13.5%.

### Outcome Measures

Primary outcomes included the mean differences in scores on the PSS-10, GAD-7, and PHQ-9 scales at the sixth week versus baseline and the changes in the prevalence rates of self-reported moderate or high stress, likely generalized anxiety disorder (GAD), and likely major depressive disorder (MDD) at the sixth week from baseline.

### Hypothesis

In a sixth-week evaluation report, 77% of subscribers to the related Text4Mood program indicated that the daily supportive text messages helped them to manage their depression and anxiety [24], which informed our decision to evaluate the Text4Hope program at the sixth week and to determine if subscribers generally had reduced anxiety and depression. Furthermore, a randomized controlled trial of daily supportive text messaging resulted in close to 25% additional improvement in mood (measured by the Beck Depression Inventory [BDI]) in the intervention group compared to the control group [23]. On this basis, we hypothesized that the Text4Hope intervention would result in >25% reduction in mean scores and prevalence rates in all 3 factors, the PSS-10, GAD-7, and PHQ-9 scales, at the sixth week versus baseline.

### Sample Size Considerations

With a projection that daily supportive text messages would result in a 25% reduction in mean PSS-10, GAD-7, and PHQ-9 scores at the sixth week from baseline, a population variance of 5.0 for each scale mean score, a one-sided significance level α=.05, and an acceptable difference between sample mean and population mean score for each scale of zero (μ – μ0 = 0), we estimated that a sample size of 686 would be sufficient to detect mean differences between the baseline and 6-week PSS-10, GAD-7, and PHQ-9 scores with a power of 80% (β=.2).

### Statistical Analysis

Data analysis was undertaken using SPSS for Windows version 26 (IBM Corporation) [33]. To assess the primary outcome measures for our intervention, we used the paired *t* test to assess the mean difference between the mean PSS-10, GAD-7, and PHQ-9 scale scores at baseline and the sixth week for subscribers who completed the instruments at both time points. In addition, we used the chi-square test to compare prevalence rates for perceived stress, likely GAD, and likely MDD at baseline and the sixth week. Moderate or high perceived stress, likely GAD, and likely MDD were assessed using cutoff scores of ≥14 on the PSS-10 [30], ≥10 on the GAD-7 [31], and ≥10 on the PHQ-9 [32], respectively. There was no imputation for missing data, and the total numbers reported represent the total responses recorded for each variable.

## Results

### Participant Demographics

Of the 766 individuals who completed both the baseline and 6-week surveys, 73 (9.6%) identified as male, 678 (88.7%) identified as female, and 13 (1.7%) identified as other gender. Table 1 provides the distribution of the demographic characteristics by gender of subscribers who completed both the baseline and sixth week surveys. Table 1 summarizes the demographic characteristics of the respondents who completed both the baseline and 6-week surveys as n (%). The data presented in Table 1 suggest that the majority of the respondents were aged between 26 and 60 years (601/758, 79.3%), White (656/766, 85.9%), had a postsecondary education (678/764, 88.7%), were employed (555/764, 72.6%), were married, cohabiting, or partnered (507/763, 66.4%), and owned homes (521/760, 68.6%).

**Table 1:** Demographic characteristics of respondents who completed both surveys by identified gender (N=766), n (%). Note that some category totals do not sum to N due to incomplete data.

Variable		Male	Female	Other	Total
**Age (years)**					
	≤25	5 (6.9)	53 (7.9)	5 (38.5)	63 (8.3)
	26-40	17 (23.6)	207 (30.8)	6 (46.2)	230 (30.3)
	41-60	33 (45.8)	337 (50.1)	1 (7.7)	371 (48.9)
	60	17 (23.6)	76 (11.3)	1 (7.7)	94 (12.4)
**Ethnicity**					
	White	55 (75.3)	590 (87.0)	11 (84.6)	656 (85.9)
	Indigenous	1 (1.4)	16 (2.4)	0 (0)	17 (2.2)
	Asian	4 (5.5)	18 (2.7)	0 (0)	22 (2.9)
	Other	13 (17.8)	54 (8.0)	2 (15.4)	69 (9.0)
**Education**					
	Less than high school diploma	5 (6.8)	14 (2.1)	1 (7.7)	20 (2.6)
	High school diploma	7 (9.6)	53 (7.8)	1 (7.7)	61 (8.0)
	Postsecondary education	61 (83.6)	606 (89.4)	11 (84.6)	678 (88.7)
	Other education	0 (0)	5 (0.7)	0 (0)	5 (0.7)
**Employment status**					
	Employed	52 (71.2)	496 (73.2)	7 (53.8)	555 (72.6)
	Unemployed	10 (13.7)	79 (11.7)	2 (15.4)	91 (11.9)
	Retired	9 (12.3)	58 (8.6)	1 (7.7)	68 (8.9)
	Student	2 (2.7)	33 (4.9)	3 (23.1)	38 (5.0)
	Other	0 (0)	12 (1.8)	0 (0)	12 (1.6)
**Relationship status**					
	Married, cohabiting, or partnered	49 (67.1)	452 (66.8)	6 (46.2)	507 (66.4)
	Separated or divorced	6 (8.2)	64 (9.5)	1 (7.7)	71 (9.3)
	Widowed	2 (2.7)	16 (2.4)	0 (0)	18 (2.4)
	Single	15 (20.5)	137 (20.2)	6 (46.2)	158 (20.7)
	Other	1 (1.4)	8 (1.2)	0 (0)	9 (1.2)
**Housing status**					
	Own a home	49 (67.1)	465 (69.0)	7 (53.8)	521 (68.6)
	Living with family	7 (9.6)	56 (8.3)	3 (23.1)	66 (8.7)
	Renting	16 (21.9)	147 (21.8)	2 (15.4)	165 (21.7)
	Other	1 (1.4)	6 (0.9)	1 (7.7)	8 (1.1)

### Outcome Measures

Table 2 presents the changes in primary outcome measures after 6 weeks from baseline for subscribers who completed both the baseline and 6-week surveys. The data displayed in Table 2 indicate that for subscribers who completed both the baseline and 6-week surveys, the mean scores on the PSS-10 and GAD-7 scales were significantly lower at 6 weeks compared to the mean scores at baseline, suggesting improvement in stress and anxiety symptoms. The effect size as measured by Cohen *d* was small (<0.5) for both the stress and anxiety scales.

There was a reduction in the mean score on the GAD-7 scale of 19.0% at the sixth week compared to the baseline scores. The reduction in the PSS-10 scores at six weeks compared to the baseline scores, although statistically significant, was much smaller (4.1%). There was no statistically significant within-subjects difference between the baseline and sixth week PHQ-9 mean scores (*P*>.05).

**Table 2:** Comparison of the baseline and 6-week mean scores on the PSS-10, GAD-7, and PHQ-9 scales for subscribers who completed both the baseline and sixth week surveys (N=766).

Measure	Responses, n^a^	Scores			Mean difference (95% CI)		*P* value		*t* value		Effect size (Cohen d)	
		Baseline score, mean (SD)	Six-week score, mean (SD)	Change from baseline, %								
PSS-10^b^	684	20.35 (6.7)	19.51 (7.0)	4.1	–0.83 (0.42 to 1.24)		<.001		3.99		0.2	
PHQ-9^c^	630	8.94 (6.0)	8.74 (5.8)	2.2	–0.20 (–0.17 to 0.57)		.28		1.08		0.2	
GAD-7^d^	612	9.62 (5.6)	7.82 (5.2)	18.7	–1.80 (1.44 to 2.16)		<.001		9.86		0.4	

^a^Not all subscribers completed all three scales; therefore, n for each scale is less than the total N.

^b^PSS-10: 10-Item Perceived Stress Scale.

^c^PHQ-9: Patient Health Questionnaire-9.

^d^GAD-7: Generalized Anxiety Disorder–7.

Table 3 indicates that there were statistically significant reductions in the prevalence rates of moderate or high stress and likely GAD but not of likely MDD when comparing the baseline and 6-week assessments. The largest reduction in prevalence rates was for anxiety (13.5%).

To assess the generalizability of our data, based on the mental health burden in our baseline samples, we examined the clinical parameters between people who only responded to the baseline survey versus those who responded to both surveys (baseline and sixth week) (Table 4 and Table 5). No statistical difference was elicited between the two groups (all *P*>.05), suggesting that at baseline, the mental health burden was similar between our study sample and subscribers who did not complete the 6-week survey.

**Table 3:** Comparison of the baseline and 6-week prevalence of moderate or high stress, likely generalized anxiety disorder, and likely major depressive disorder.

Condition	Prevalence, n/total responses (%)		Change in prevalence rate (sixth week from baseline), %	χ^2^ (df)	*P* value
	Baseline	Sixth week			
Moderate or high stress^a^	642/748 (85.8)	582/742 (80.4)	–5.4	7.78 (1)	.01
Likely major depressive disorder^b^	288/723 (39.8)	262/688 (38.1)	–1.7	0.46 (1)	.50
Likely generalized anxiety disorder^c^	326/712 (45.8)	220/682 (32.3)	–13.5	26.76 (1)	<.001

^a^Assessed using a cutoff score of ≥14 on the 10-Item Perceived Stress Scale.

^b^Assessed using a cutoff score of ≥10 on the Patient Health Questionnaire-9.

^c^Assessed using a cutoff score of ≥10 on the Generalized Anxiety Disorder-7.

**Table 4:** Comparison of the prevalence rates of moderate or high stress, likely generalized anxiety disorder, and likely major depressive disorder between subscribers who only completed the baseline survey and subscribers who completed both the baseline and 6-week surveys.

Condition	Prevalence rate at baseline, n/total responses (%)		χ^2^ (df)	*P* value
	Subscribers who completed the baseline assessment but not the 6-week assessment	Subscribers who completed both the baseline and 6-week assessments		
Moderate or high stress	4065/4798 (84.7)	642/748 (85.8)	0.62 (1)	.43
Likely major depressive disorder	1848/4447 (41.6)	288/723 (39.8)	0.76 (1)	.38
Likely generalized anxiety disorder	2040/4364 (46.7)	326/712 (45.8)	0.23 (1)	.63

**Table 5:** Comparison of the mean scores on the PSS-10, GAD-7, and PHQ-9 scales between subscribers who only completed the baseline survey and subscribers who completed both the baseline and 6-week surveys.

Scale	Score at baseline, mean (SD)		Independent *t* test	*P* value
	Subscribers who completed the baseline assessment but not the 6-week assessment	Subscribers who completed both the baseline and 6-week assessments		
PSS-10^a^	20.55 (6.77)	20.30 (6.71)	0.96	.34
PHQ-9^b^	9.03 (6.22)	8.94 (6.0)	0.35	.73
GAD-7^c^	9.64 (5.93)	9.56 (5.65)	0.37	.72

^a^PSS-10: 10-Item Perceived Stress Scale.

^b^PHQ-9: Patient Health Questionnaire-9.

^c^GAD-7: Generalized Anxiety Disorder–7.

Similarly, we examined the clinical parameters between subscribers who responded to the 6-week survey only and subscribers who responded to both surveys (Table 6 and Table 7). No statistical difference was elicited in prevalence of stress, anxiety, or depression symptoms between the two groups (*P*>.05), suggesting that after receiving the intervention for 6 weeks, the mental health burden was similar between our study sample and subscribers who only completed the 6-week survey.

**Table 6:** Comparison of the prevalence rates of moderate or high stress, likely generalized anxiety disorder, and likely major depressive disorder between subscribers who completed both the baseline and 6-week surveys and subscribers who only completed the 6-week survey.

Condition	Prevalence rate at sixth week, n/total responses (%)		χ^2^ (df)	*P* value
	Subscribers who completed the 6-week assessment but not the baseline assessment	Subscribers who completed both the baseline and 6-week assessments		
Moderate or high stress	1217/1518 (80.2)	582/724 (80.4)	0.01 (1)	.91
Likely major depressive disorder	483/1378 (35.1)	262/688 (38.1)	1.83 (1)	.18
Likely generalized anxiety disorder	430/1361 (31.6)	220/682 (32.3)	0.09 (1)	.76

**Table 7:** Comparison of the mean scores on the PSS-10, GAD-7, and PHQ-9 between subscribers who completed both the baseline and 6-week surveys and subscribers who only completed the 6-week survey.

Scale	Score at sixth week, mean (SD)		Independent *t* test	*P* value
	Subscribers who completed the sixth-week assessments but not the baseline assessments	Subscribers who completed both the baseline and sixth week assessments		
PSS-14^a^	19.36 (7.12)	19.44 (7.05)	–0.25	.80
PHQ-9^b^	8.20 (5.79)	8.69 (5.75)	–1.79	.07
GAD-7^c^	7.55 (5.40)	7.71 (5.21)	–0.66	.51

^a^PSS-10: 10-Item Perceived Stress Scale.

^b^PHQ-9: Patient Health Questionnaire-9.

^c^GAD-7: Generalized Anxiety Disorder–7.

## Discussion

### Principal Findings

The Text4Hope program was provided as an intervention tool for the general population to support the mental well-being of individuals living in the Canadian province of Alberta during the global COVID-19 pandemic. Other technology-based interfaces have been deployed during the COVID-19 pandemic to track the disease spread in populations [34], to gather data related to the general knowledge, attitudes, and behavior of the public related to the pandemic [35,36], and to offer mental health support to the public during the pandemic [37-40]. To the best of our knowledge, this is the first study to assess the impact of a text-messaging intervention on self-reported symptoms of stress, anxiety, and depression experienced during the COVID-19 pandemic. Our study yielded interesting results regarding temporal changes in the self-reported severity and rates of symptomatology related to the three psychiatric health conditions under study. After receiving daily messages for 6 weeks, we observed significant reductions in the respondents’ mean scores on the GAD-7 (18.7%) and the PSS-10 (4.0%), suggesting that the program was effective in reducing anxiety and stress symptomatology in the respondents. There was no significant reduction in the mean PHQ-9 score at 6 weeks from baseline. In terms of prevalence rates, the largest significant reduction in prevalence rate was for likely GAD (13.5%), followed by moderate or high stress (4.1%). Again, there was no significant reduction in the prevalence rate of likely MDD at 6 weeks from baseline.

The self-reported rates of anxiety symptoms in our study at baseline were higher than those reported in other studies [41,42]. However, the greatest improvement recorded after the provision of the Text4Hope program was for anxiety, with a 13.5% reduction in symptom prevalence rate and 19% improvement in GAD-7 score. Text4Hope achieved a small Cohen *d* effect size (0.4) in mitigating anxiety symptoms, which is comparable to the effect sizes found for an internet CBT program aimed at reducing anxiety symptoms [43]. Typically, interventions that do not include therapist support demonstrate lower effect size outcomes compared to those including therapists [44,45]. By contrast, our intervention reached thousands of individuals during the pandemic, and it aimed to provide a general population intervention rather than individual psychotherapy. The overall change in GAD-7 scores in our study (–1.8) appears to be consistent with the magnitude of score changes recorded after providing other remote health services. For example, adding a telephone service to computerized CBT in combating anxiety yielded a reduction of 1.18 in GAD-7 scores [46]. Again, the percentage of change in GAD-7 scores in our study after providing a daily text message for 6 weeks (19.0%) is consistent with the effect of medications on anxiety symptoms; in a very large randomized controlled trial in the United Kingdom, sertraline was evidently effective in reducing GAD-7 scores by 21% after 6 weeks, and this result was described as clinically important [47].

Our findings indicate that there was a modest effect of the program on improving stress symptoms, with greater benefit than other internet-based cognitive behavioral theory (iCBT) platforms [48]. In a Japanese study, iCBT was used to alleviate anxiety, stress, and depressive symptoms in university students [48]. The most significant effect was mainly reported for anxiety, while stress symptoms did not show a difference between case and control group members after the intervention period [48].

There was no significant change between baseline and 6-week mean scores for likely MDD. Comparing our results with other remotely delivered health services yielded variable results. Two meta-analyses found that the effectiveness of iCBT programs, including MoodGYM, in mitigating depressive symptoms showed small effect sizes, especially in short-term assessments [43,44,49]. On the other hand, a significant improvement in BDI-II score with moderate effect size was observed at 3 months in patients with depression and comorbid alcohol use disorder who received supportive SMS text messages twice daily compared to the control group, who only received a thank-you SMS text message every fortnight [23]. The Text4Hope program was primarily designed as a health promotion tool to support the general population in Alberta during the COVID-19 pandemic and to combat potential stress and anxiety symptoms that are usually associated with epidemics or global crises. Our study participants were members of the general population rather than a patient sample, which may account for the observed differences in results. Furthermore, in a previous randomized trial [23], participants received the intervention for 12 weeks compared to 6 weeks in our study, which may account for the observed differences in the effects. Another study examined the effect of sertraline, an antidepressant of the selective serotonin reuptake inhibitor class, in ameliorating depressive symptoms; this study reported only a 5% relative reduction (95% CI 7%-15%; *P*=.41) in the mean PHQ-9 score at week 6 [47], which is not vastly different from the apparent 2% improvement observed with our Text4Hope intervention.

Three months after the launch of the Text4Hope program, the dropout rate was 13.5%. A high withdrawal rate is not uncommon for a texting service provided via SMS. When Bendsten and Bendsten [50] compared an SMS texting service to services provided via email, they found that people in the SMS group opted out at significantly higher rates than those in the email group (20.1% versus 5.2%, respectively). Additionally, in a review study on behavioral changing interventions provided via SMS text message, authors reported a wide range of withdrawal rates (0%-57%) among participants. [51]. They justified this result as being due to the untailored and unilateral nature of these texting programs, which may be less engaging and therefore may result in low retention rates [51].

### Limitations of the Study

Our study has several limitations. For ethical reasons, we lacked a comparative control group that did not receive the Text4Hope intervention during the same phase of the pandemic against which the recorded changes in stress, anxiety, and depression levels could be compared. It is therefore possible that the reductions in stress, anxiety, and depression levels are not all attributable to the Text4Hope intervention. Second, we relied on self-rated scales to assess stress, anxiety, and depression symptomatology, which could potentially overestimate the levels of these mental disorders when compared with prevalence rates that would have been obtained using structured clinical interviews with the *Diagnostic and Statistical Manual of Mental Disorders 5th Edition*. Third, our results may not be generalizable to the general population and are at risk of participation bias, where individuals with pre-existing mental health conditions are characteristically more inclined to enroll in the Text4Hope program compared with individuals with no pre-existing mental health disorders. We did not ask subscribers about pre-existing mental disorders, which would have helped to distinguish new symptoms from pre-existing ones but may have resulted in limited enrolment; in our experience, subtle changes in signup processes for subscribers can result in marked decreases in participation. Finally, the sample size of subscribers who completed both the baseline and 6-week assessments was rather small, and it is possible that other subscribers had variable changes in their mental health parameters from baseline to the sixth week. This limitation notwithstanding, our sample size was larger than the projected 686 subscribers needed to detect mean differences between the baseline and 6-week PSS-10, GAD-7, and PHQ-9 scores with a power of 80%. In addition, the mental health burden in our sample at baseline and at the sixth week were not significantly different from those of subscribers who only completed the baseline survey and the subscribers who completed the 6-week survey, respectively (Tables 4 and 5). Furthermore, the demographic characteristics of subscribers who completed both the baseline and 6-week surveys mirror those of all 8267 subscribers who completed the baseline survey by July 12, 2020 [52]. Specifically, the proportions of the various demographic characteristics in our sample compared to the proportions of the same demographics in the larger sample of 8267 subscribers was as follows: female gender, 88.7% versus 87.1%, respectively; White, 85.9% versus 82.3%, respectively; postsecondary education, 88.7% versus 85.2%, respectively; employed, 72.6% versus 73.3%, respectively; married, cohabiting, or partnered, 66.4% versus 71.1%, respectively; and homeowner, 68.6% versus 65.9%, respectively [52]. These proportions support the generalizability of our results to all subscribers. Future studies using this style of intervention could attempt to minimize attrition by offering incentives for participation or by sending messages encouraging people to continue subscribing.

Finally, the effect sizes in our study were relatively small, which may minimize the strength of the produced results. However, interventions that do not include therapists often report low effect sizes compared to those including therapists [44,45].

### Conclusion

The Text4Hope program resulted in statistically significant reductions in mean scores on the PSS-10 and GAD-7 scales but not the PHQ-9 scale at the sixth week from baseline. The program also resulted in statistically significant reductions in subscribers’ prevalence rates of moderate or high stress and likely GAD but not of likely MDD. The largest reductions in the mean scores and prevalence rates were observed for anxiety symptoms. It should be noted this paper reports data from the midpoint of the Text4Hope program implementation, and the rates of change for outcomes for stress, anxiety, and depression may differ after the program ends at 3 months.

The relatively large improvements in anxiety symptoms achieved in our sample after 6 weeks of receiving the intervention during the COVID-19 pandemic suggest that the Text4Hope program is a useful intervention that can be deployed during natural and humanitarian disasters to support individuals at the population level. Over half of Canadians have reported that their mental health needs are not fully met [53]. A commonly reported reason is the cost of services [53]. As such, free mobile-based services such as Text4Hope can help address financial barriers. In the Canadian context and in other global contexts, these services can also be delivered remotely, which helps maintain essential physical distancing requirements during pandemics but also provides a means of access for those at rural or remote locations with little or no capacity for accessing mental health support services. We did not differentiate urban from rural or remote subscribers, as the program was offered to everyone in the province; however, regardless of geographical region, the pattern of urban, rural, and remote subscribers’ locations is of interest in relation to understanding the full value of SMS text messaging in this context. This is an additional step that we are considering in evaluating this program approach.

## Abbreviations

BDI: Beck Depression Inventory
CBT: cognitive behavioral therapy
GAD: generalized anxiety disorder
GAD-7: Generalized Anxiety Disorder–7
iCBT: internet-based cognitive behavioral therapy
MDD: major depressive disorder
PHQ-9: Patient Health Questionnaire–9
PSS-10: 10-Item Perceived Stress Scale
